# Supplementary material for: Population genomic analyses reveal a highly differentiated and endangered genetic cluster of northern goshawks (Accipiter gentilis laingi) in Haida Gwaii
Source: Evol Appl. 2019 Jan 12;12(4):757–72. doi: 10.1111/eva.12754 (PMC6439496; doi:10.1111/eva.12754)
Supplement: Supplementary file 1 [file EVA-12-757-s001.pdf]

Supporting Figures for:

Geraldes, A.*, K.K. Askelson*, E. Nikelski, F.I. Doyle, W.L. Harrower, K. Winker, and D.E. Irwin. Population genomic analyses reveal a highly differentiated and endangered genetic cluster of northern goshawks (*Accipiter gentilis laingi*) in Haida Gwaii. ***Evolutionary Applications***, in press (accepted 12 December 2018). (*Shared first authorship)

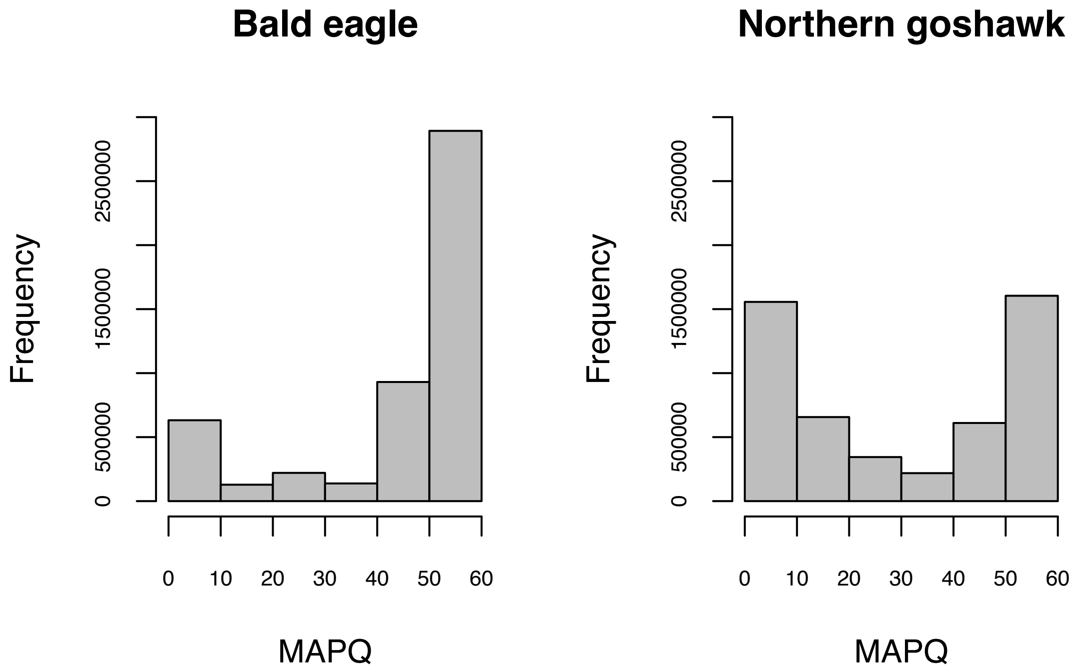

Supporting Figure 1. Comparison of read mapping quality to the bald eagle genome for reads from a bald eagle (on the left, total read number 4.94 M) and a northern goshawk (NGAK020, on the right, total read number 4.99 M). MapQ is $-10\log_{10}$Pr(mapping is wrong), e.g., reads with MaqQ below 20 have a probability of being wrongly mapped of 0.05 or higher.

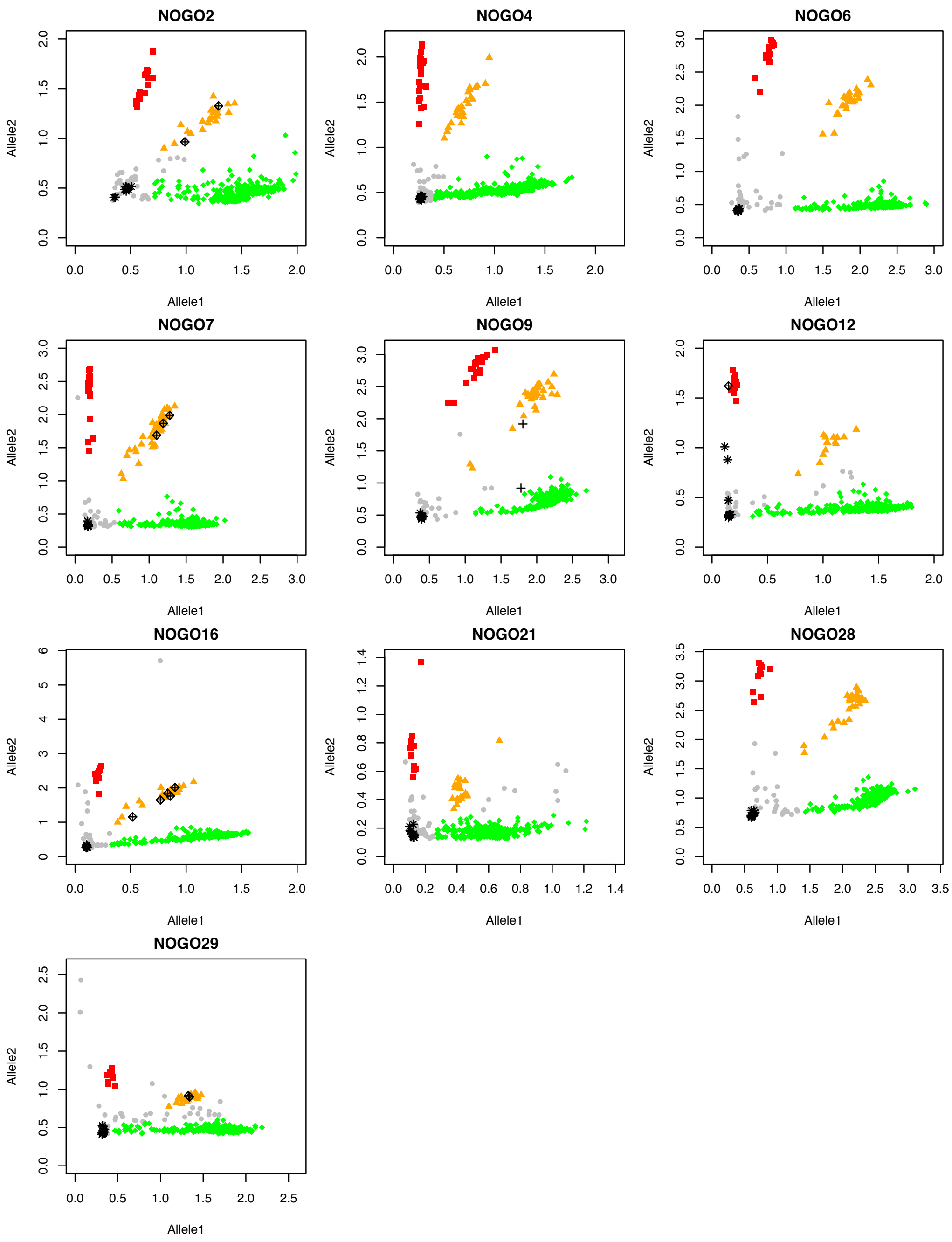

Supporting Figure 2. Genotyping results and allelic discrimination for 10 TaqMan assays. Axes show the ΔRn values of each allele. Red squares indicate homozygous genotypes for Allele 2, green diamonds indicate homozygous genotypes for Allele 1, orange triangles indicate heterozygous genotypes, gray circles indicate failed genotypes, asterisks indicate negative controls, crossed diamonds indicate discrepancies between GBS and TaqMan genotypes and plus signs indicate TaqMan discrepancies (samples genotyped in both plates with different inferred genotypes; these were considered failed). See Supporting Table 6 for individual genotype calls.

A)

B)

C)

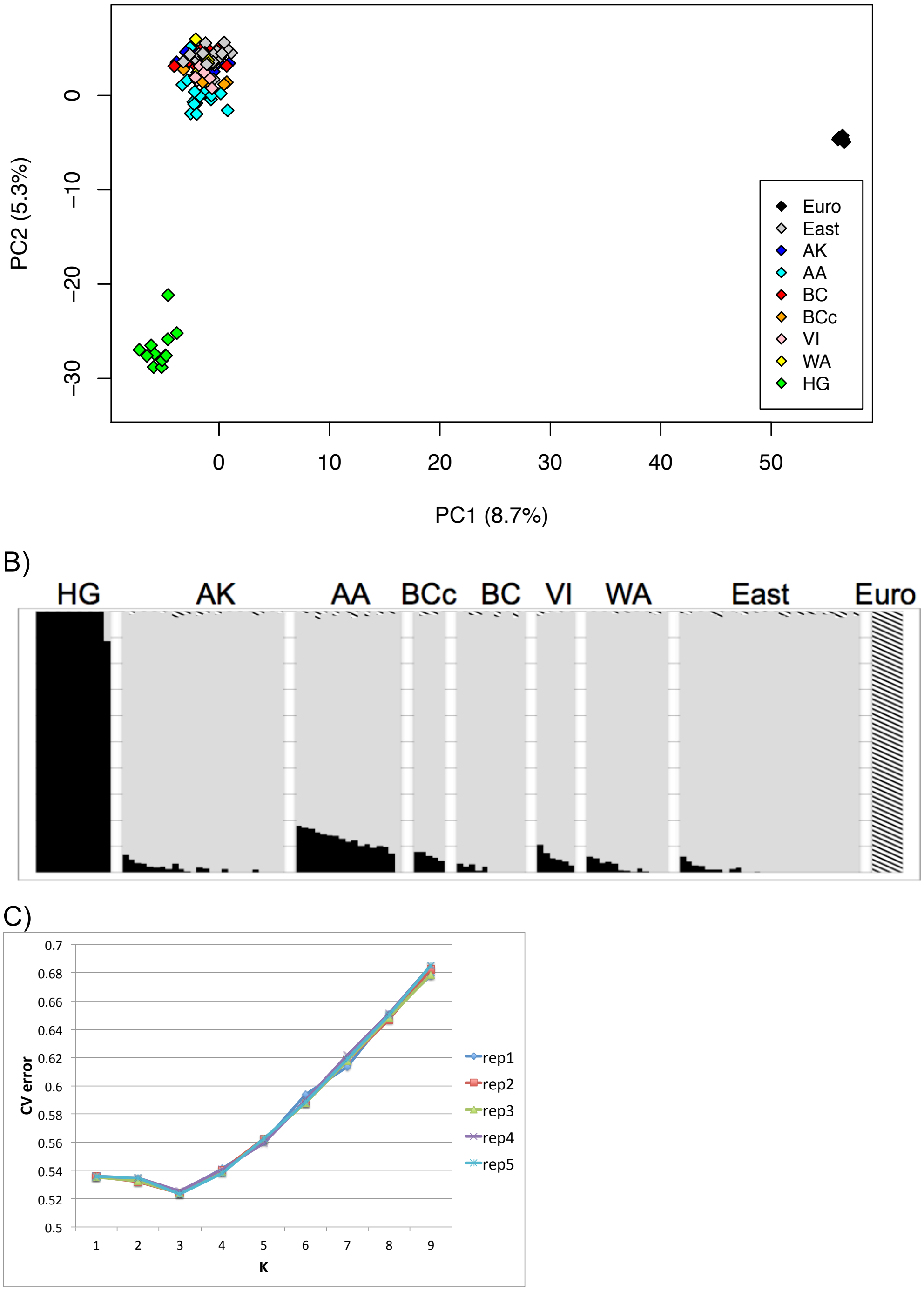

Supporting Figure 3. Analyses of population structure among northern goshawks sampling regions inferred with the GBS nuclear data for 124 samples (*n* = 6,006 unlinked SNPs with minor allele frequency of 0.05 or higher). Population names and sample sizes can be found in Table 1. A) Principal components analysis. B) Admixture analysis for *K* = 3, the value of *K* that minimizes the cross-validation error as shown in C.

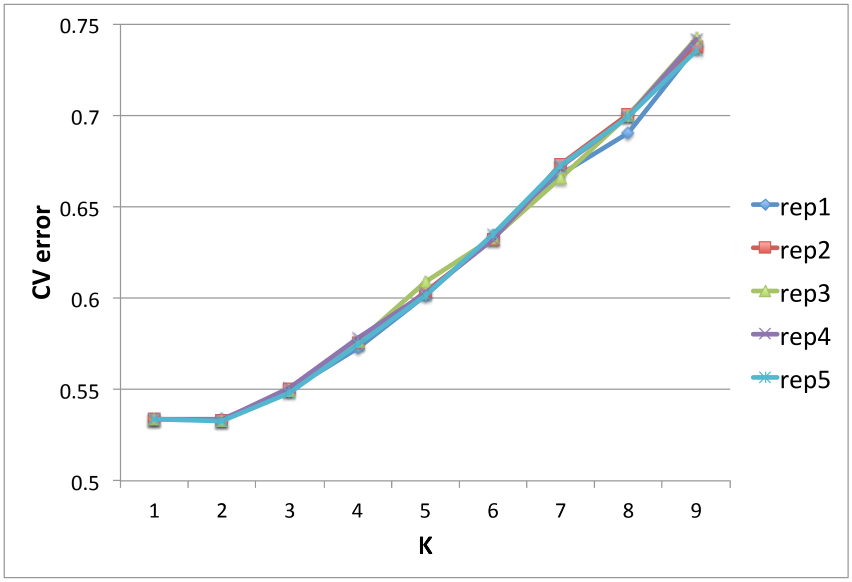

Supporting Figure 4. Cross validation error analysis for Admixture analysis with 119 northern goshawks from North America using 6,058 unlinked SNPs with minor allele frequency of 0.05 or higher. *K* = 2 minimizes the cross-validation error, but the cross-validation error for *K* = 1 is almost as low.

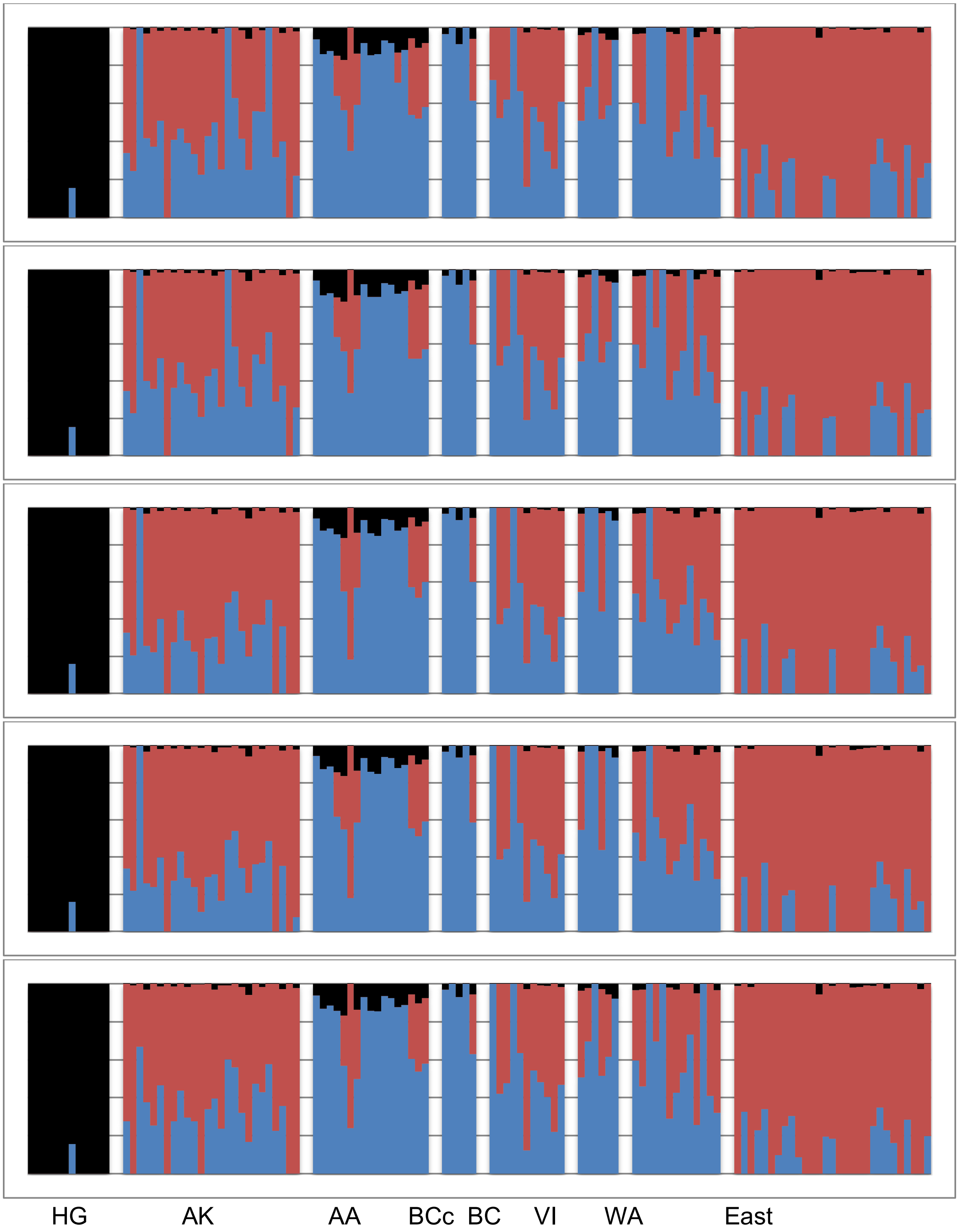

Supporting Figure 5. Admixture results for 119 North American goshawks based on 6,058 unlinked SNPs with minor allele frequency of 0.05 or above at K = 3. All five replicates are shown.

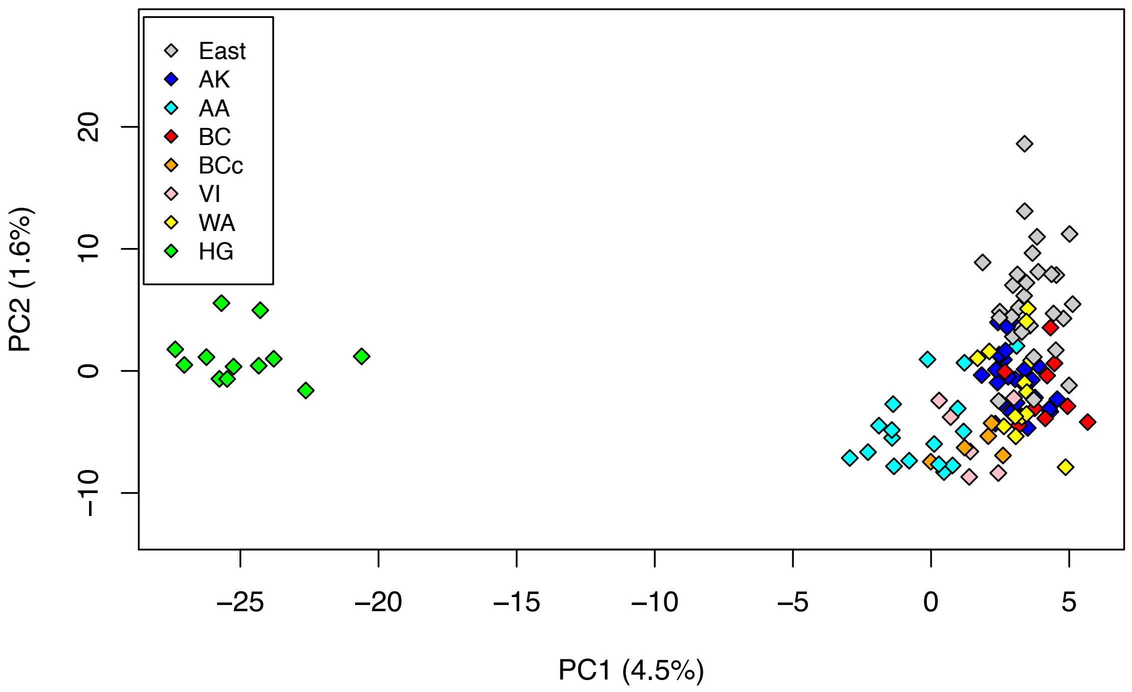

Supporting Figure 6. Principal Components Analysis of population structure in 119 northern goshawk samples from North America, based on 24,322 unlinked SNPs (singletons excluded).

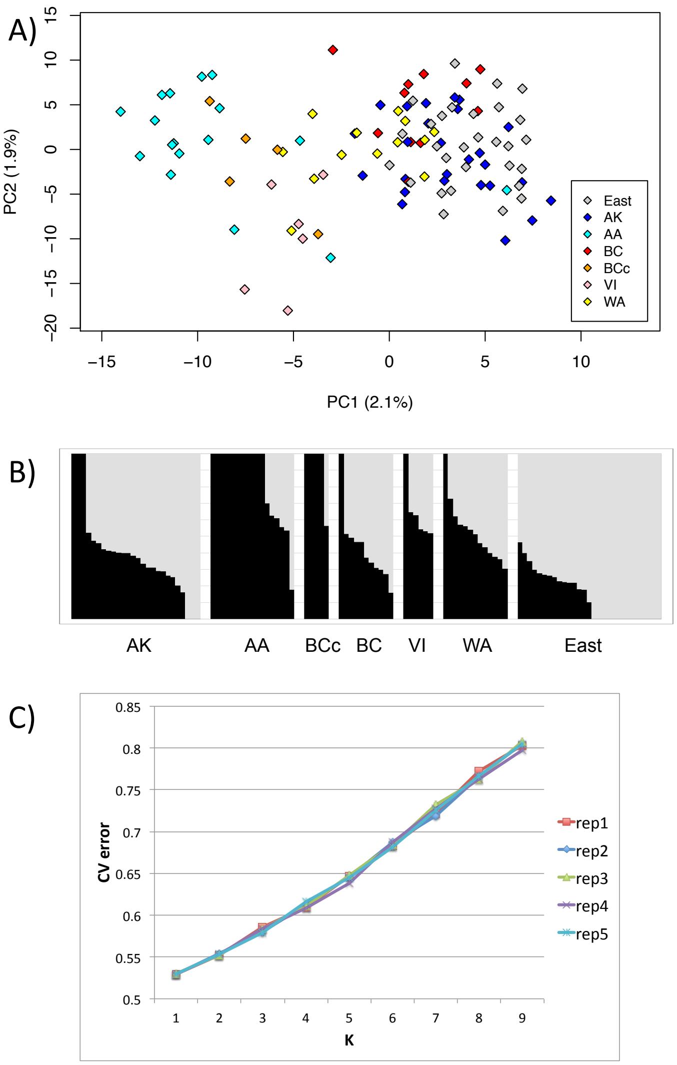

Supporting Figure 7. Analyses of population structure among northern goshawks sampled from North America, excluding Haida Gwaii, inferred from GBS nuclear data from 107 samples (*n* = 6,297 unlinked SNPs with minor allele frequency of 0.05 or higher). Population names and sample sizes can be found in Table 1. A) Principal components analysis, B) Admixture analysis for *K* = 2, and C) cross validation error analysis across all 5 replicates. Note that although *K* = 2 is plotted in B, *K* = 1 is the value that minimizes the cross validation error.

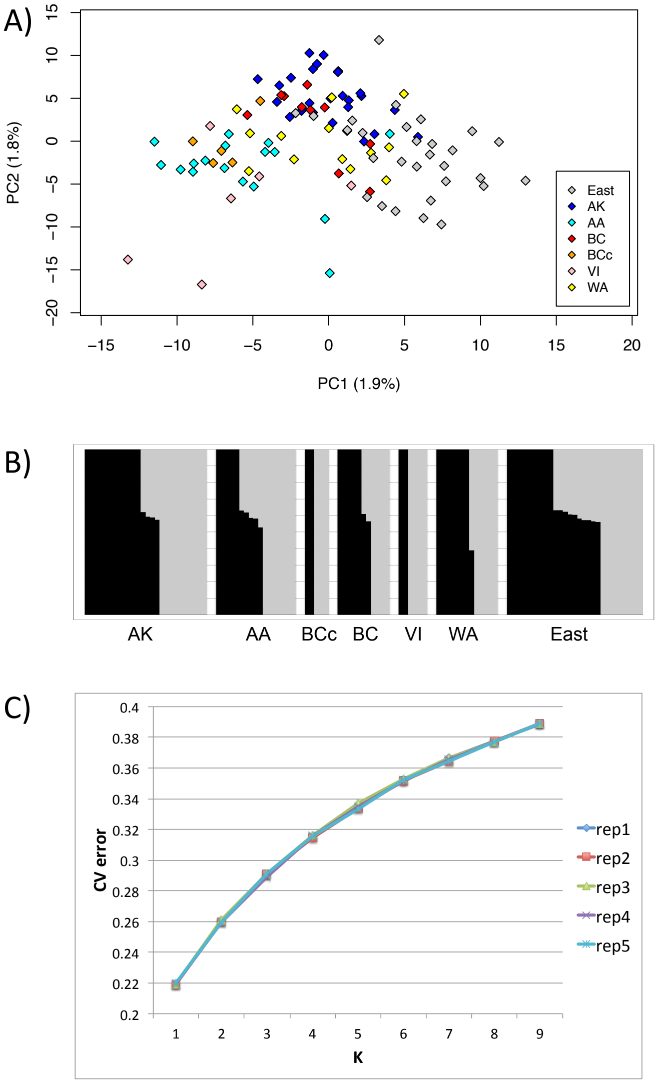

Supporting Figure 8. Analyses of population structure among northern goshawks sampled in North America, excluding Haida Gwaii, inferred from the GBS nuclear data for 107 samples (*n* = 22,611 unlinked SNPs with singletons excluded). Population names and sample sizes can be found in Table 1. A) Principal components analysis, B) Admixture analysis for *K* = 2 and C) cross validation error analysis across all 5 replicates. Note that although *K* = 2 is plotted in B, *K* = 1 is the value that minimizes the cross validation error.

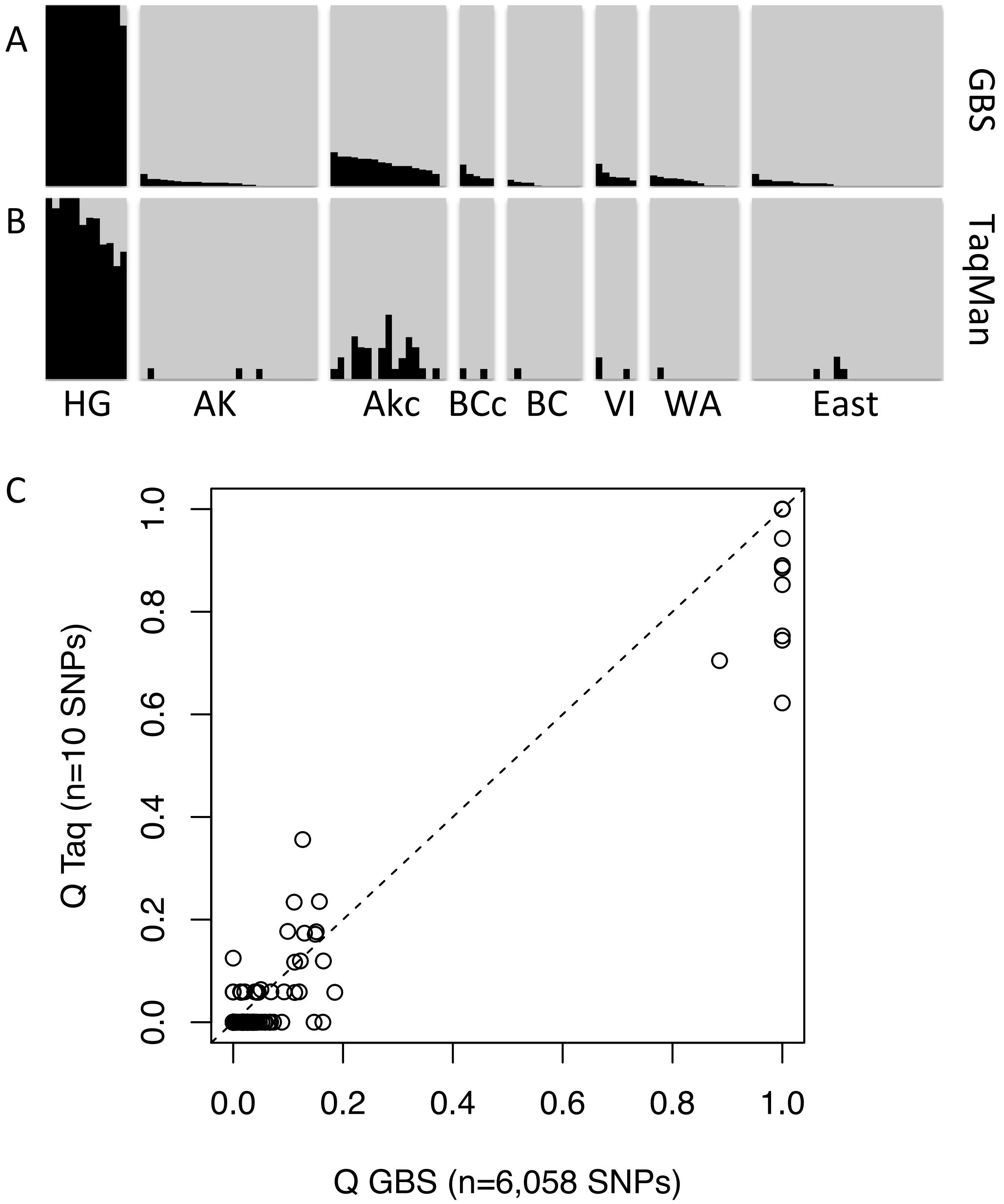

Supporting Figure 9. Comparison of genomic admixture estimates for 118 samples for which both GBS and TaqMan data was available. A) Admixture's Q estimates (*K* = 2 with 6,058 SNPs unlinked SNPs with minor allele frequency of 0.05 or higher) and Admixture's Q estimates (*n* = 10 TaqMan loci). C) Data in A and B plotted in the X and Y axes. Dotted line indicates where samples would fall if they had the same Q estimate from both datasets.

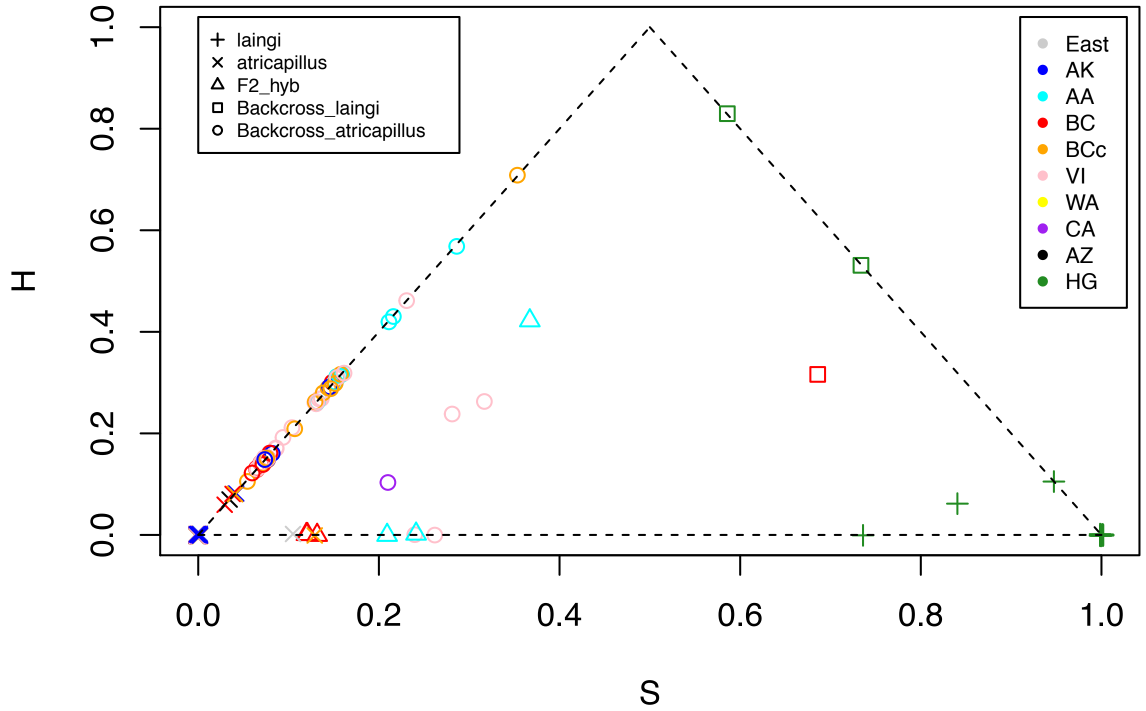

Supporting Figure 10. Plot of ancestry index (*S*) and interclass heterozygosity (*H*) for 386 North American northern goshawks genotyped at 10 loci that are ancestry informative. Different symbols indicate the highest likelihood estimation of the hybrid class of each sample. Note that some samples have very high and close likelihood of being in different categories, e.g. HG sample RC06Z01 has *S* = 0.58 and *H* = 0.83 and has the highest likelihood for the hybrid class backcross to the *laingi* cluster (log-likelihood = -6.64), but the likelihood of it being an F1 hybrid (log-likelihood=-6.71) is almost as high. See Supporting Table 6 for complete results. Note that in this figure, "laingi" refers to the HG genetic cluster, whereas "atricapillus" refers to genetic cluster common everywhere outside of HG.

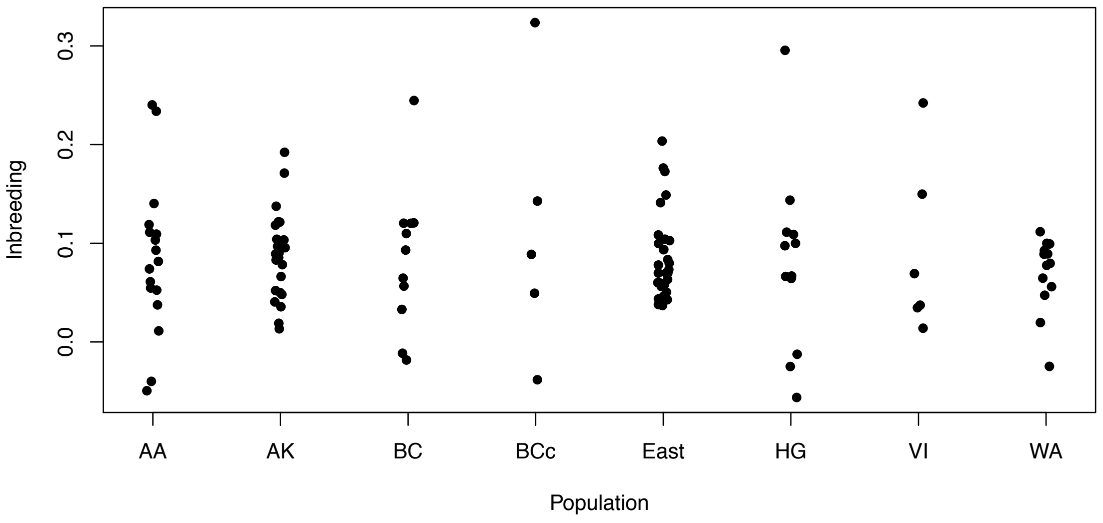

Supporting Figure 11. Estimates of the average genome-wide inbreeding coefficient, for each individual within each population. A negative F indicates that there is an excess of observed heterozygote SNPs (and suggests outbreeding), while positive values indicate a deficit of observed heterozygotes (and suggests inbreeding). Individual values can be found in Supporting Table 11.
