# Supplementary material for: Population genomic analyses reveal a highly differentiated and endangered genetic cluster of northern goshawks (Accipiter gentilis laingi) in Haida Gwaii
Source: Evol Appl. 2019 Jan 12;12(4):757–72. doi: 10.1111/eva.12754 (PMC6439496; doi:10.1111/eva.12754)
Supplement: Supplementary file 3 [file EVA-12-757-s003.pdf]

Supplemental File 1 for:

Geraldes, A.*, K.K. Askelson*, E. Nikelski, F.I. Doyle, W.L. Harrower, K. Winker, and D.E. Irwin. Population genomic analyses reveal a highly differentiated and endangered genetic cluster of northern goshawks (*Accipiter gentilis laingi*) in Haida Gwaii. ***Evolutionary Applications***, in press (accepted 12 December 2018). (*Shared first authorship)

Supplemental file 1 contents:

i. Identification of putatively sex-linked loci (page 2)
ii. Estimation of pairwise kinship coefficients (page 12)
iii. References (page 13)

i) Identification of putatively sex-linked loci

During initial exploratory analysis of the GBS dataset, we performed a PCA analysis with the package SNPrelate in R (Zheng et al. 2012), on 99 *A. gentilis* samples from the Pacific Northwest using 2,474 unlinked (using default LD pruning in the R package SNPrelate) biallelic SNPs, with less than 30% missing genotypes, genotype quality above 10, minor allele frequency of 0.1 or above (VCFtools v0.1.11 [Danecek et al. 2011] commands “--max-alleles 2”, “--min-alleles 2”, “--remove-indels”, “--max-missing 0.7”, “--minGQ 10”, “--maf 0.1”), and observed heterozygosity below 0.6 (with a custom perl script from Owens et al., 2016). PCA1 explained 4.89% of the variance in the data and separated Haida Gwaii samples from other North American samples, while PCA2 explained 2.23% of the variance and created two clusters of samples with no discernable geographic pattern (Supplemental Figure 1A). When samples were identified by the sex reported by the sample collectors (either as Male, Female or Unknown/Juvenile), it became apparent that PCA2 was separating samples based on sex, except for six samples reported as female that clustered with males (QL22Z29, QL22Z24, QK01Z05, QI01Z05, QL22Z34, QI01Z11) and one sample reported as male that clustered with females (QK01Z04; Supplemental Figure 1B).

Supplemental Figure 1A

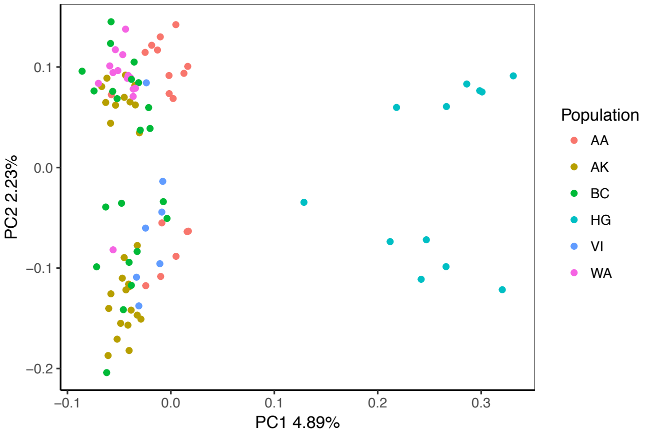

Supplemental Figure 1B

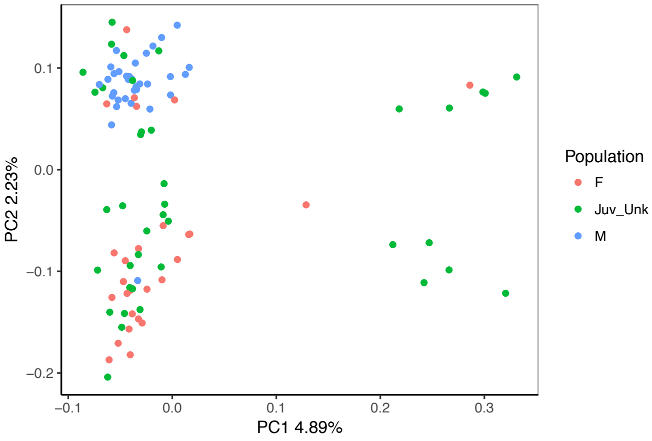

Supplemental Figure 1. PCA on 99 samples from the Pacific Northwest based on 2,474 unlinked SNPs with MAF ≥ 0.1. PCA1 separates HG from other PNW samples (Figure 1A), and PCA2 separates samples mostly by sex (Figure 1B).

This suggested that some loci may be sex linked and that they are driving the pattern in PCA2 which separates samples based on their sex. To determine whether PCA2 in fact separates samples by sex, we attempted to determine the sex of 128 samples (Supporting Table 1) with a sex determination PCR assay (Ito et al. 2003). We followed Ito et al. (2003) to perform ARMS-PCR amplifications with three primers in a 30 µl final volume including 1 µM of primer P2 and 0.5 µM of primers NP and MP, 0.2 mM of dNTP's, 1.5 mM of $MgCl_2$, 1 unit of *Taq* DNA polymerase, recombinant (Invitrogen), and up to 100 ng of DNA. Cycling conditions were: 94°C for 3 minutes, 38 cycles of 94°C for 30 seconds, 51°C for 45 seconds, 72°C for 35 seconds and a final extension step at 72°C for 10 minutes. PCR products were visualized under UV after gel electrophoresis in 1X TAE at 75 V for 45 minutes in a 2.5% (w/v) agarose gel stained with SYBR Safe (Invitrogen). Female samples produced two PCR bands and male samples one. The assay was successful for 109 samples and failed for 19 samples. Of the 99 samples in the PCA, 83 were successfully genotyped with the Ito et al. (2013) assay, and there were no disagreements in the inferred sex from the PCR assay and PCA2 (this includes six of the seven samples above with discrepancy between the sex reported by collectors and position on the PCA; one sample failed with the Ito et al. (2013) PCR assay). This confirms that PCA2 separates samples by sex.

Again, the analyses above suggest that in our dataset some loci are sex linked. Note that our reads were mapped to the Bald eagle reference genome, which is from a male bird, and therefore no W loci are expected.

To determine which loci are the best candidates for being sex linked we assembled a GBS SNP dataset with a subset of 58 northern goshawk samples (from the 99 above) from North America for which no population structure was expected (excluding Haida Gwaii samples and samples where the inferred sex from all three methods did not match [Supporting Table 1]). This 58 sample dataset (34 males and 24 females) was filtered as above, except that we now accepted sites with observed heterozygosity of 0.75 or lower instead of 0.6, resulting in 11,723 SNPs. We performed a PCA with 5,195 unlinked SNPs after trimming the data for LD with SNPrelate in R. PCA1 explains 4.42% of the variation and corresponds to sex (24 females have negative values and 34 males positive values; Supplemental Figure 2A) and PCA2 explains 2.33% of the variation and mostly separates the Alexander Archipelago samples from others (Supplemental Figure 2B).

The complete dataset of 11,723 SNPs was used to a) estimate F_{ST} between male and female samples, b) perform a genome wide association study (GWAS) with sex as the phenotype, and c) perform a GWAS where the phenotype is the position of each sample along the first axis of the PCA with 5,195 unlinked SNPs (Supplemental Figure 2A).

Supplemental Figure 2A

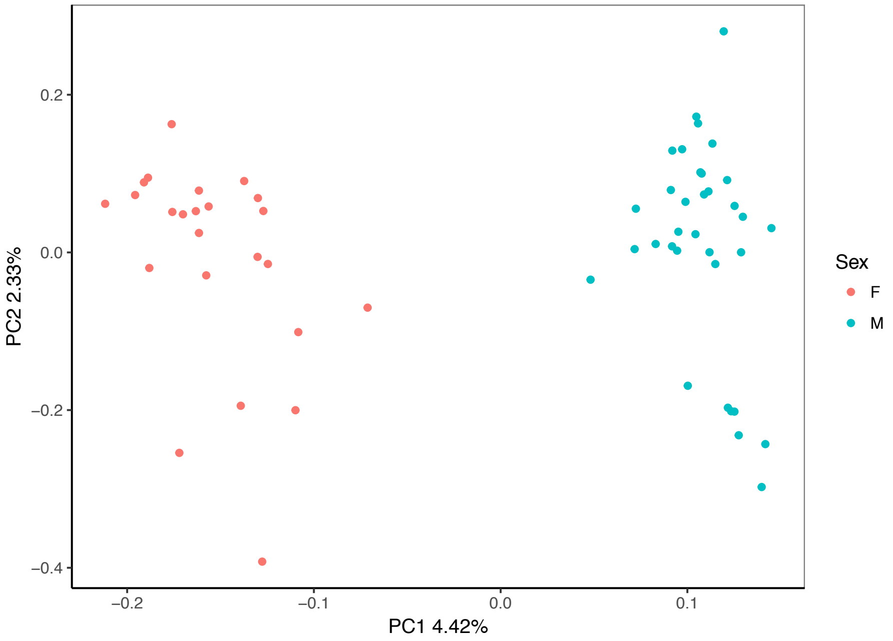

Supplemental Figure 2B

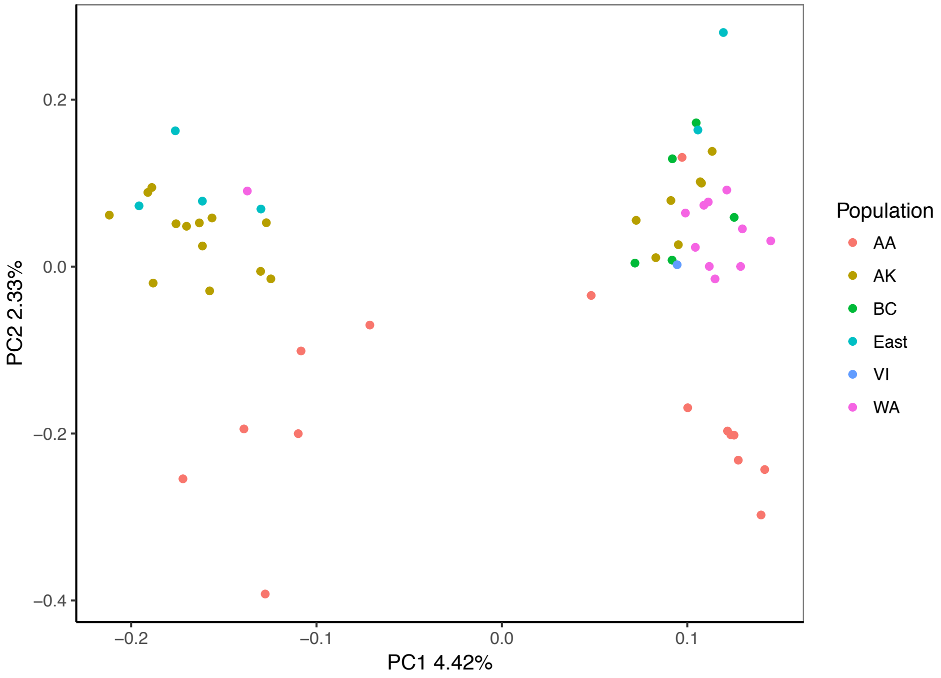

Supplemental Figure 2. PCA based on 5,195 SNPs for 58 North American northern goshawk samples (excluding Haida Gwaii) of known sex (34 males and 24 females), with dots colored according to (A) sex or (B) sampling region.

a) F_{ST} between 34 males and 24 females

We calculated the F_{ST} between males and females for each SNP in this dataset. We expected that sex linked SNPs would show high F_{ST} between males and females while

non sex linked SNPs would have an F_{ST} close to zero in this mostly unstructured population (as seen in Supplemental Figure 2B, there is some differentiation between samples from the Alexander Archipelago and others). We calculated F_{ST} in VCFtools with the command "--weir-fst-pop". We also calculated the F_{ST} between the Alexander Archipelago (AA, n = 15) and the remaining samples (n = 43). The weighted F_{ST} estimate between sexes is 0.0375 and the weighted F_{ST} between AA-others is 0.0067. For each SNP we then took the difference between the F_{ST} for sex and the F_{ST} for populations (Sex-Pop F_{ST}).

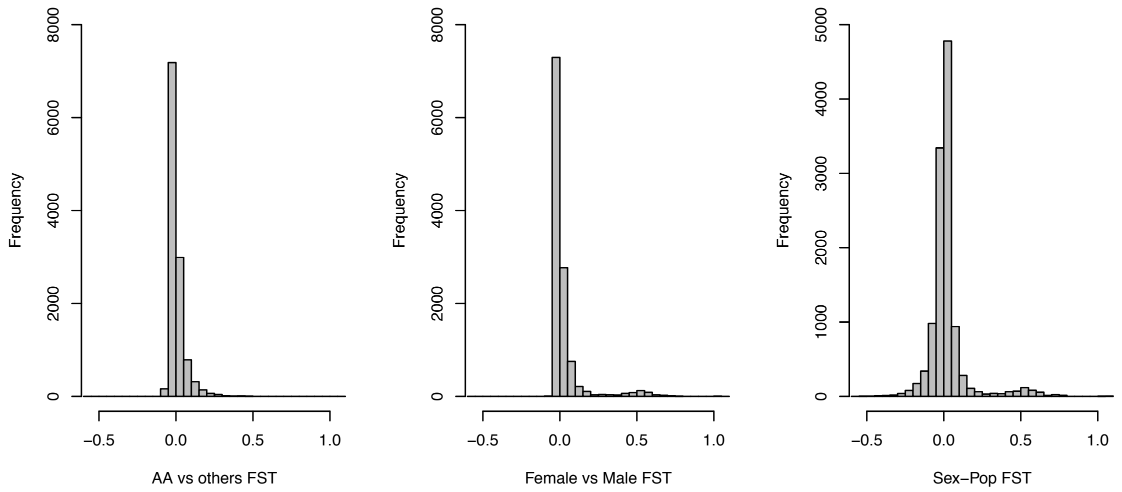

Supplemental Figure 3. Histograms of F_{ST}, for a set of 58 samples and 11,723 SNPs, from left to right, between Alexander Archipelago and other populations (AA vs others), between Females and Males (Females vs Males) and for each locus the difference between the F_{ST} between Males and Females and the F_{ST} between Populations (Sex-Pop). In the rightmost panel, SNPs that are unrelated to sex are centered on 0 and SNPs with a strong sex effect are shifted towards high values. We took those with Sex-Pop F_{ST} of 0.25 or higher as candidates for being sex linked.

The largest values of Sex-Pop F_{ST} should correspond to sex linked SNPs (Supplemental Figure 3). Sex-Pop was 0.25 or above for 552 SNPs. A PCA, calculated as above, but without these 552 SNPs revealed no separation of samples by sex (Supplemental Figure 4A), and PCA1 still mostly separated samples from the Alexander Archipelago (Supplemental Figure 4B).

Supplemental Figure 4A

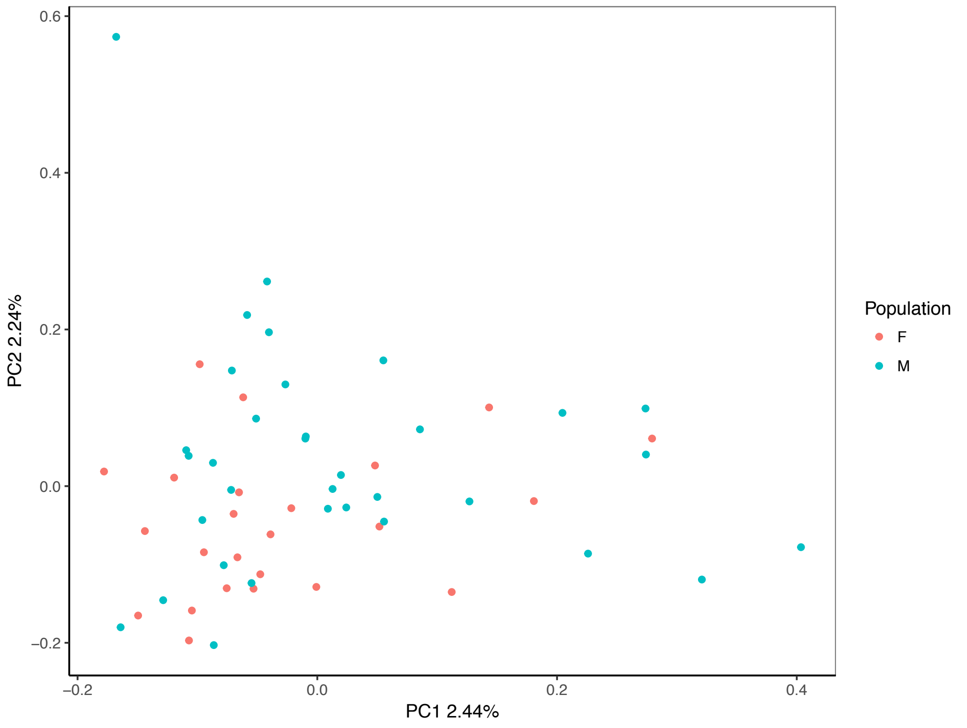

Supplemental Figure 4B

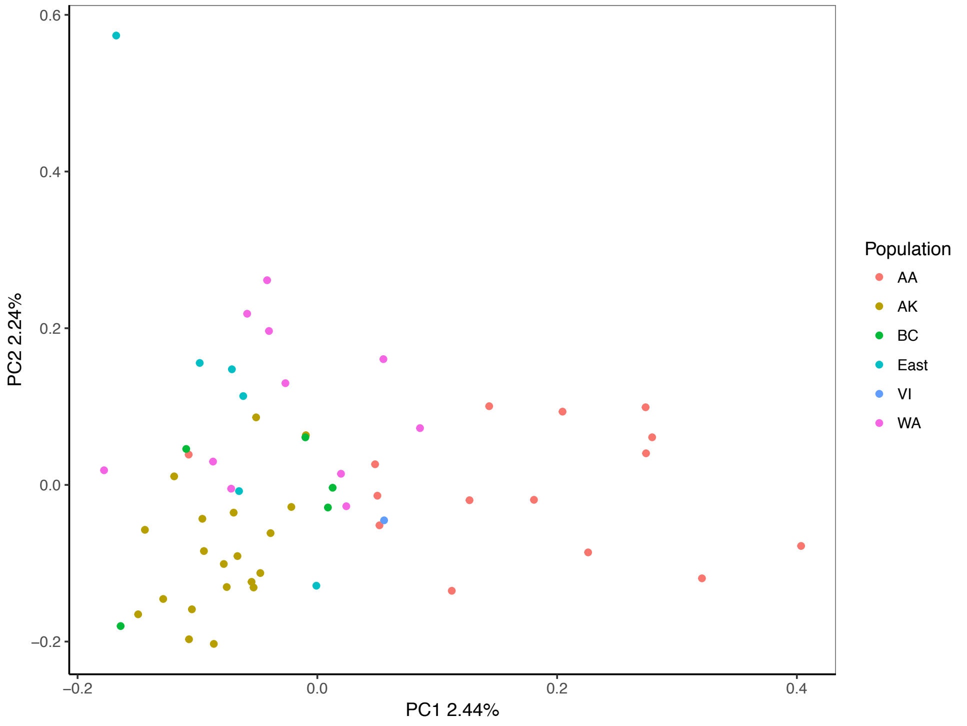

Supplemental Figure 4. PCA plots eliminating 552 SNPs with Sex-Pop F_{ST} and Population F_{ST} higher than 0.25. After LD pruning 5,215 SNPs were used. A) Plotted by sex and B) plotted by population.

b) GWAS with sex as discrete phenotype

We used the VCFtools command “--plink” to generate an input file for Plink (Chang et al. 2015) for the 11,723 SNP dataset, where we performed a simple case-control GWAS (“--assoc --adjust" commands in Plink) where 34 males were coded as cases and 24 females were coded as controls. There were 458 SNPs associated with sex ($\alpha < 0.001$ after false discovery rate correction for multiple testing [Benjamini and Yekutieli 2001] [Supplemental Figure 5]).

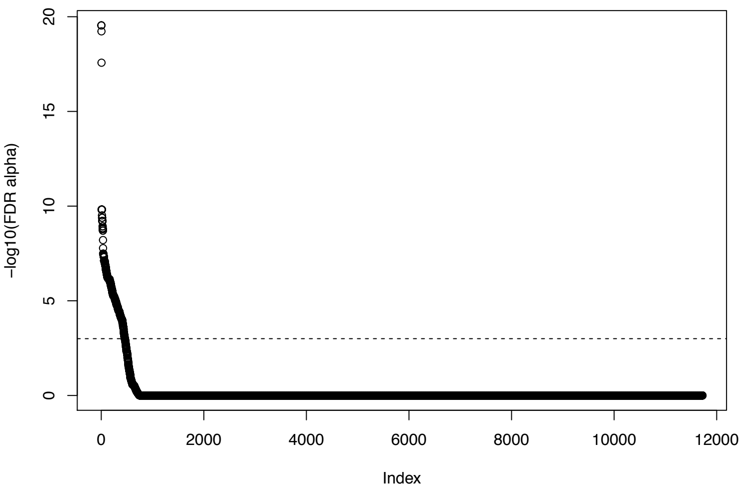

Supplemental Figure 5. Plot of the distribution of corrected *p*-values for a GWAS between sex and genotype. Dashed line corresponds to a $\alpha = 0.001$ after false discovery rate correction for multiple testing. 458 SNPs are above the dashed line and are therefore statistically significantly associated with sex.

Supplemental Figure 6A

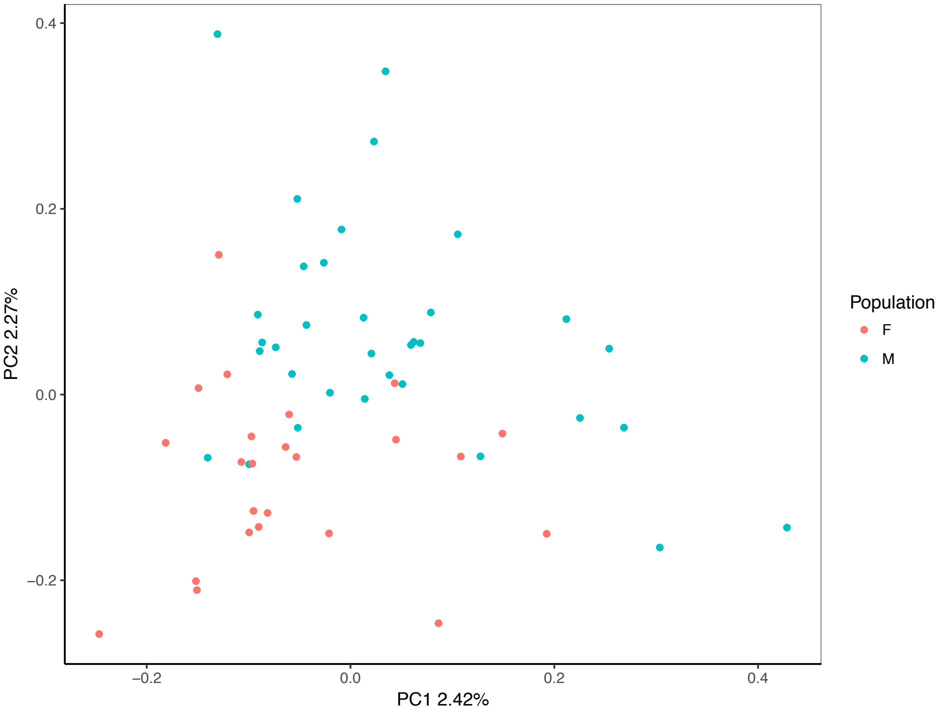

Supplemental Figure 6B

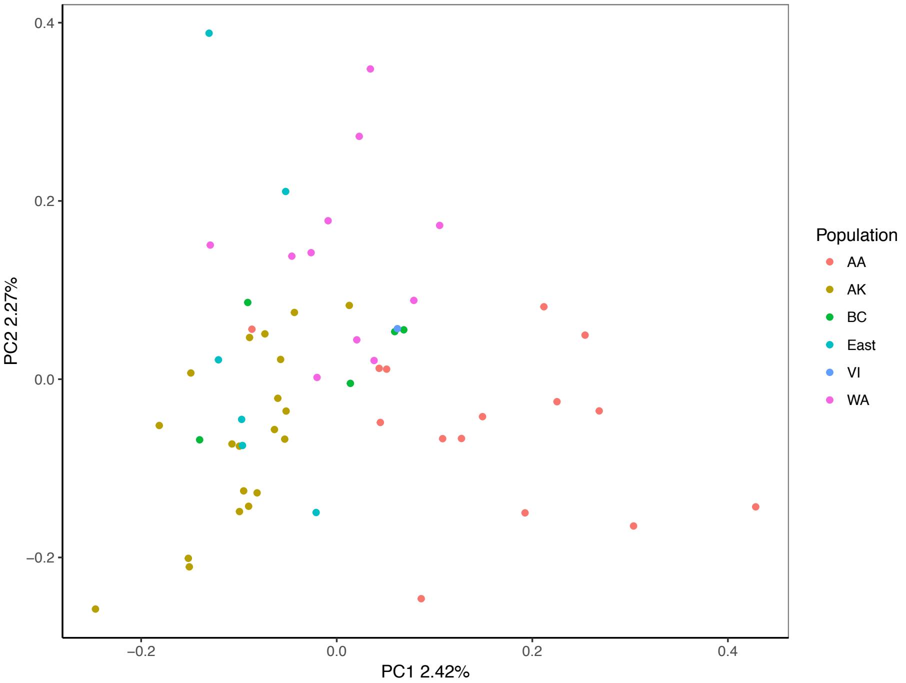

Supplemental Figure 6. PCA plots based on 5,135 SNPs after LD pruning and elimination of 458 SNPs that are significant in a GWAS where the phenotype is sex at $\alpha < 0.001$ after FDR. A) Plotted by sex and B) plotted by population.

A PCA, calculated as above, but without these 458 SNPs revealed no separation of samples by sex (Supplemental Figure 6A), and PCA1 still mostly separated samples from the Alexander Archipelago (Supplemental Figure 6B).

c) GWAS with position along PCA1 as quantitative phenotype

We used the same Plink file as in b) but with the phenotype being the position along the first axis of the PCA in Supplemental Figure 2. There were 486 SNPs associated with position along the first axis of the PCA ($\alpha < 0.001$ after false discovery rate correction for multiple testing [Benjamini and Yekutieli 2001] [Supplemental Figure 7]).

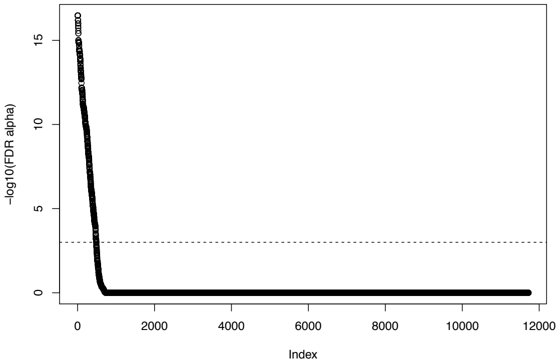

Supplemental Figure 7. Plot of the distribution of corrected *p*-values for a GWAS between position along PCA1 and genotype. Dashed line corresponds to a $\alpha = 0.001$ after false discovery rate correction for multiple testing. 486 SNPs are above the dashed line and are therefore statistically significantly associated with sex (or position along PCA1).

Supplemental Figure 8A

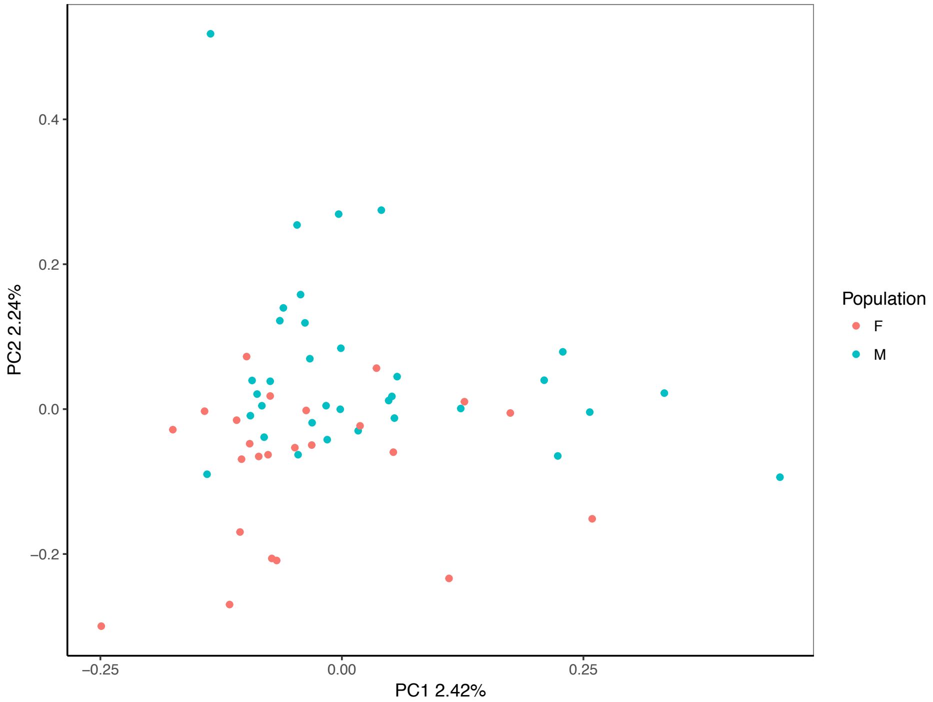

Supplemental Figure 8B

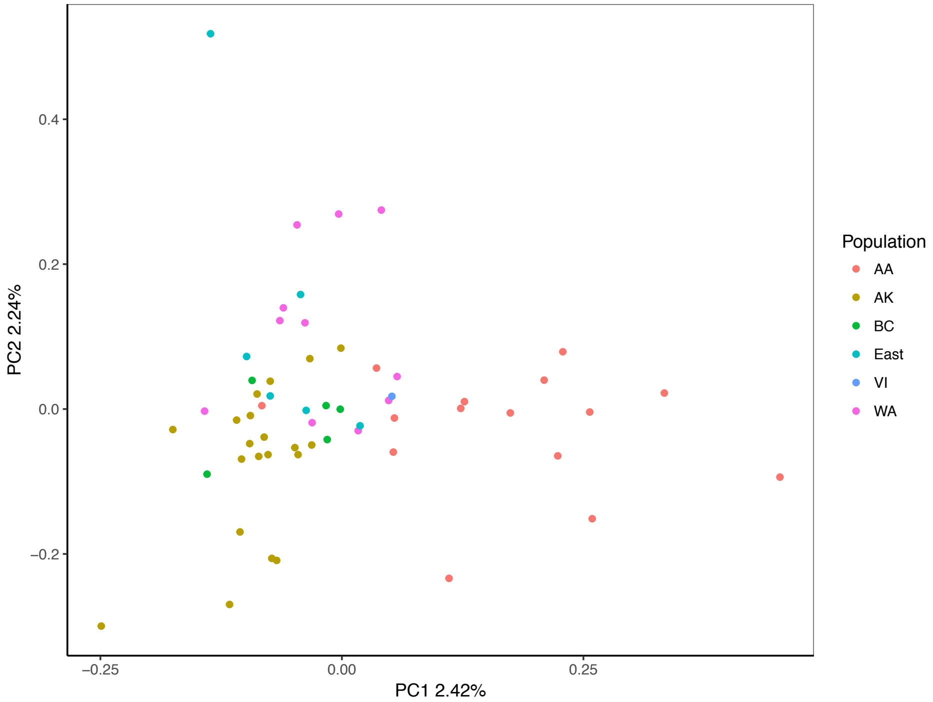

Supplemental Figure 8. PCA plots based on 5,136 SNPs after LD pruning and elimination of 486 SNPs that are significant in a GWAS where the phenotype is position along PCA1 of Supplemental Figure 2, a proxy for sex, at α < 0.001 after FDR. A) Plotted by sex and B) plotted by population.

Overlap between approaches

The results from the three different approaches showed extensive overlap. There were 554 SNPs identified by at least one of these three approaches. Of those, 444 SNPs were identified by all three approaches, 54 by only two and 56 by only one. We consider

all 554 SNPs identified by at least one approach as a strong candidate for being sex linked. We used VCFtools to obtain the allele frequency in the entire sample of 58 individuals, and also the allele frequencies and observed genotypes for each sex for all 554 SNPs. This information can be found in Supporting Table 2. Three patterns emerge for the segregation of these SNPs. For a putative A to T polymorphism:

Pattern	Male genotypes	Female genotypes
ZW divergence	AA	AT
W polymorphism	AA	AA/AT
Z polymorphism	AA/AT/TT	AA/TT

As the bald eagle reference sequence is that of a male, no W sequences were expected. We interpret the patterns of ZW divergence and W polymorphism as being caused by the mapping to a distantly related reference sequence where sequences from northern goshawk W linked genes are being mapped to their Z orthologs in bald eagle. We eliminated all 554 SNPs above from any further analysis in this manuscript.

ii) Estimation of pairwise kinship coefficients

To determine if any pairs of samples in our dataset had close familial relationships, we estimated kinship coefficients for two datasets independently, a) all samples from Haida Gwaii (*n* = 12; 5,204 SNPs) and b) all other northern goshawk samples from North America (*n* = 114; 6,260 SNPs). The samples used in each dataset can be found in Supporting Table 1. For each dataset, SNPs were filtered so that only unlinked SNPs (LD pruning performed in the R package SNPrelate with "method=r" and "ld.threshold=0.2") with less than 30% missing genotypes, genotype quality above 10, minor allele frequency of 0.05 or above (VCFtools v0.1.11 [Danecek et al. 2011] commands "--max-alleles 2", "--min-alleles 2", "--remove-indels", "--max-missing 0.7", "--minGQ 10", "--maf 0.05"), and observed heterozygosity below 0.6 (with a custom perl script from Owens et al., 2016) were included. Kinship coefficients were calculated in the R package SNPrelate with function IBDKING and default parameters. No close familial relationships were found in the Haida Gwaii dataset (Supporting table 3). For the 114 sample dataset, 8 kinship estimates corresponded to second degree kinship or higher (Supporting Table 4). Samples QF01Z26 and QF01Z01 were classified as duplicates and QF01Z01 was removed; samples QF01Z12 and QF01Z02 were classified as 2nd-degree kin and QF01Z02 was removed; QF01Z13 and QF01Z03 were classified as 2nd-degree kin and QF01Z03 was removed; QL22Z26 and Ql22Z31 were classified as 2nd-degree kin and QL22Z31 was removed; QI01Z05 and QI01Z04 were classified as 2nd-degree kin and QI01Z05 was removed; and finally, three samples (QF01Z10, QF01Z11, QF01Z14) from one nest in the interior of British Columbia were classified as 2nd-degree kin and two (QF01Z10, QF01Z11) were removed. All 12 samples from Haida Gwaii and 107 samples from other regions of North America remained for further analyses.

References

Benjamini, Y., & Yekutieli, D. (2001). The control of the false discovery rate in multiple testing under dependency. *Annals of Statistics*, *29*, 1165–1188. https://www.jstor.org/stable/2674075

Chang, C. C, Chow, C. C., Tellier, L. C. A. M., Vattikuti, S., Purcell, S. M., Lee, J. J. (2015). Second-generation PLINK: rising to the challenge of larger and richer datasets. *GigaScience*, *4*, 7. https://doi.org/10.1186/s13742-015-0047-8

Danecek, P., Auton, A., Abecasis, G., Albers, C. A., Banks, E., DePristo, M. A., et al. (2011). The variant call format and VCFtools. *Bioinformatics*, *27*, 2156–2158. http://doi.org/10.1093/bioinformatics/btr330

Ito, H., Sudo-Yamaji, A., Abe, M., Murase, T., & Tsubota, T. (2003). Sex identification by alternative polymerase chain reaction methods in Falconiformes. Zoological Science, 20, 339–344. https://doi.org/10.2108/zsj.20.339

Owens, G. L., Baute, G. J., & Rieseberg, L. H. (2016). Revisiting a classic case of introgression: hybridization and gene flow in Californian sunflowers. *Molecular Ecology*, *25*, 2630–2643. http://doi.org/10.1111/mec.13569

Zheng, X., Levine, D., Shen, J., Gogarten, S., Laurie, C., & Weir, B. (2012). A high-performance computing toolset for relatedness and principal component analysis of SNP data. *Bioinformatics*, 28, 3326–3328. https://doi.org/10.1093/bioinformatics/bts606
